# Prediction of the Risk of Laparoscopy-Assisted Gastrectomy by Comparing Visceral Fat Area and Body Mass Index

**DOI:** 10.1155/2018/1359626

**Published:** 2018-09-13

**Authors:** Yongke Liu, Dong Guo, Zhaojian Niu, Yuliang Wang, Guanghua Fu, Yanbing Zhou, Qingkai Xue, Xinliang Jin, Zhiqi Gong

**Affiliations:** ^1^Department of General Surgery, The Affiliated Hospital of Qingdao University, Medical College of Qingdao University, No. 16 Jiangsu Rd, Qingdao City, China; ^2^Department of General Surgery, The Affiliated Hospital of Qingdao University, No. 16 Jiangsu Rd, Qingdao City, China

## Abstract

**Propose:**

The purpose of this study was to compare the accuracy of visceral fat area (VFA) and body mass index (BMI) in predicting the risk of laparoscopic-assisted gastrectomy.

**Methods:**

Clinicopathological and imaging data of 133 patients who underwent laparoscopy-assisted gastrectomy were recorded, including 17 cases of conversion to open surgery. The remaining 116 patients were retrospectively analyzed after we excluded 17 patients who had been transferred to laparotomy. The patients were divided into two groups according to BMI (≤25 kg/m^2^: BMI-L group; >25 kg/m^2^: BMI-H group) and VFA (≤100 cm^2^: VFA-L group; >100 cm^2^: VFA-H group). Clinical outcomes were compared between the BMI and VFA subgroups.

**Results:**

There were no differences in intraoperative blood loss and the number of harvested lymph nodes between low and high patients defined by BMI and VFA (*p* > 0.050). However, in the comparison of patients who underwent laparoscopic resection only, it was found that the operation time and intraoperative blood loss of the VFA-H group were more than those of the VFA-L group (*p* < 0.050). Compared to the VFA-L group, the VFA-H group had later first exhaust time (*p* = 0.018), more complications (*p* < 0.001), and longer hospital stays (*p* = 0.049). However, no similar conclusion was obtained in the BMI group (*p* > 0.050).

**Conclusion:**

This study demonstrates that VFA better evaluates the difficulty of laparoscopy-assisted gastrectomy and the risk of postoperative complications than BMI.

## 1. Introduction

With the extensive application of laparoscopy-assisted gastrectomy in patients with gastric carcinoma, research on the influencing factors of laparoscopy-assisted gastrectomy for gastric cancer has become increasingly widespread. Research has shown that obesity is one of the most important factors that interfere with laparoscopic surgery. Obesity can significantly increase the incidence of postoperative complications of laparoscopy-assisted gastrectomy in patients with gastric carcinoma and reduce the thoroughness of abdominal lymph node dissection; some surgeons do not recommend that obese patients undergo laparoscopic surgery [1–4]. Nevertheless, other studies have shown that when obese and nonobese patients were compared after laparoscopy-assisted gastrectomy for gastric carcinoma, there were no differences in operation times or in the incidence of conversion to open surgery and postoperative complications, and the postoperative hospital stays for obese patients were similar or even shorter than those for nonobese patients [4, 5]. Consequentially, it is valuable to explore the influence of obesity on laparoscopic gastrectomy in patients with gastric carcinoma. BMI has been widely used clinically as a measure of the degree of obesity in patients with indicators, but owing to age, gender, and other factors, there was a big difference between the distributions of fat in individuals; therefore, BMI may not accurately reflect the extent of visceral fat accumulation in patients [6–10]. This study found that visceral fat areas from a single scan obtained at the level of the umbilicus (the approximate level of L4 and L5) by CT can reflect the objective index of the upper abdomen as well as the lower abdominal fat reserves [11]. VFA is closely related to the total volume of visceral fat, which can be used as an evaluation index of obesity and to determine the extent of operation difficulty and the incidence of postoperative complications of open surgery [5]. Accordingly, this study was a retrospective analysis to compare the accuracy of visceral fat area (VFA) and body mass index (BMI) in evaluating the prediction of the risk of laparoscopic-assisted gastrectomy for gastric cancer.

## 2. Materials and Methods

### 2.1. Research Object

This investigation was a single-center study of 133 patients with gastric cancer who underwent laparoscopic-assisted gastrectomy between May 2011 and July 2014 at the Affiliated Hospital of Qingdao University. The remaining 116 patients were retrospectively analyzed after we excluded 17 patients who had been transferred to laparotomy. Preoperative gastroscopy and postoperative pathological examination of patients confirmed gastric cancer. No history of abdominal operation was recorded in those patients, and none were admitted to hospital 30 days after discharge. All the patients underwent multislice spiral CT (Discovery CT750, GE Healthcare, Chicago, IL, USA) scanning after preoperative fasting for 8 hours, and all of the images were transmitted to the central data system. The patients' clinicopathological and imaging data were retrospectively analyzed.

### 2.2. Grouping Method and Estimation of BMI and VFA

**Figure 1:**
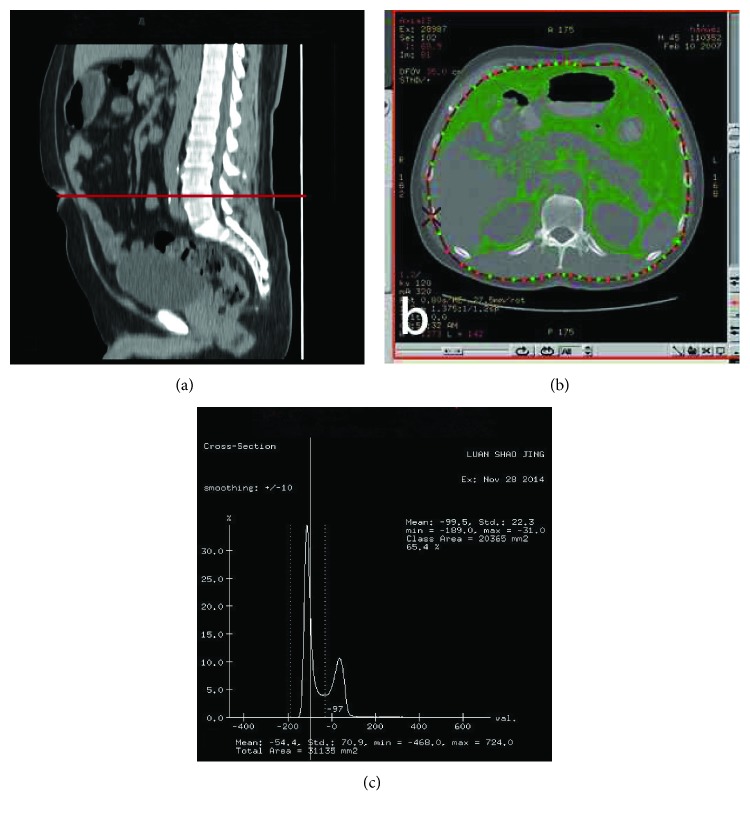
Quantification of visceral fat area: (a) select CT images at the umbilical level to calculate the visceral fat area; (b) with the mouse to drag the coil marked visceral tissue region; (c) we can determine the threshold by dragging the dotted line in the view. Adipose tissue was determined by setting the attenuation level within the range of −190 to −30 Hounsfield units. VFA was quantified automatically by the software.

All the patients were classified as obese or nonobese using both VFA and BMI criteria. BMI was calculated by weight divided by the square of the height of the body, and obese patients were defined as those with BMI > 25 kg/m^2^ (BMI-H). The remainder was classified as nonobese (BMI-L) in accordance with the criteria of the Japan Society for the Study of Obesity [10–12]. According to the results of other studies, we defined the visceral fat area greater than 100 cm^2^ as VFA-H and the remainder as VFA-L. [13] The patients were divided into two groups according to their BMI, a high BMI group (BMI > 25 kg/m^2^, *n* = 46) and a low BMI group (BMI ≤ 25 kg/m^2^, *n* = 70). The patients were also divided by VFA into two groups, a high VFA group (VFA > 100 cm^2^, *n* = 58) and a low VFA group (VFA < 100 cm^2^, *n* = 58). Histogram software was used to convert the horizontal axial image of the umbilical region to the irregular image rendering of the specified CT number, while determining the area of a specific region was defined as VFA [14–16]. The operation is shown in Figure 1.

### 2.3. Observation Measures

All intraoperative- and postoperative-related indexes of the patients were compared between the BMI and VFA subgroups for both of the definitions. The intraoperative-related indexes included the operation time, the incidence of conversion to open surgery, intraoperative blood loss, and the number of dissected lymph nodes. The postoperative correlation indexes included complications, first exhaust time, and the length of hospital stay.

### 2.4. Surgical Procedures

The surgical group consisted of an operator, a first assistant surgeon, and one endoscopist. The operating surgeon independently completed 60 cases of laparoscopic-assisted gastrectomy of gastric cancer each year and directed the procedures. The operative procedures included laparoscopic-assisted total and distal gastrectomy, proximal gastrectomy, and gastrojejunostomy. Postoperative routine nutritional support and gastrointestinal surgery medications were also assessed.

### 2.5. Statistical Analysis

All the statistical data were analyzed using SPSS 17.0 software. The count data in the two groups were compared using the *χ*^2^ test or Fisher's exact probability test and composition ratio representation for the count data. The Mann–Whitney *U* test and *χ*^2^ test were used to compare the measurement data in the two groups, which were expressed as mean ± standard deviation (mean ± SD). Finally, *p* < 0.050 was considered statistically significant.

## 3. Results

### 3.1. Distribution of VFA and BMI

**Figure 2:**
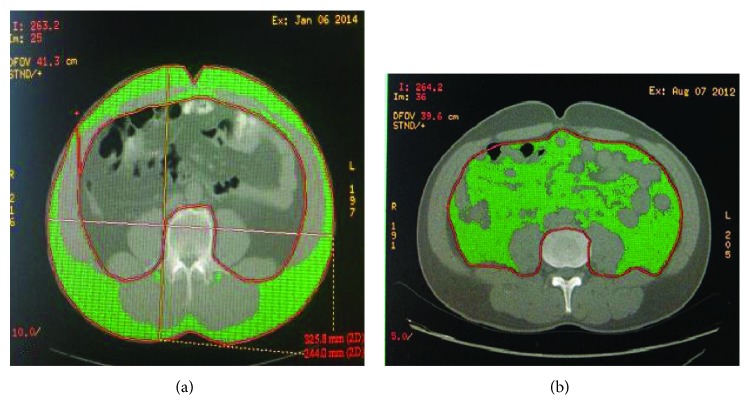
Images demonstrating the method used to determine abdominal fat, distribution on a CT scan obtained at the navel level: (a) the green area represents subcutaneous fat; (b) the green area represents visceral fat area.

There were 70 (60.34%) cases in the BMI-L group, 46 (39.66%) cases in the BMI-H group, 58 (50.00%) cases in the VFA-H group, and 58 (50.00%) cases in the VFA-L group. There were 19 cases in the VFA-H group, and 7 BMI-H patients were VFA-L. BMI and VFA were used to identify the differences between obesity and nonobesity. This means that, according to BMI, the obese patients were not necessarily those with visceral obesity.  Figure 2 shows the patients with similar BMI but with visceral fat and subcutaneous fat.

### 3.2. Patient Characteristics

Patient characteristics and other information are shown in Tables 1 and 2. There was no significant difference in gender, operation mode, and location of the tumor between the BMI and VFA subgroups, respectively (*p* > 0.050). The two groups had statistically significant differences in age. This may be associated with increasing age at the time of abdominal adipose tissue accumulation. Because most of the patients with gastric cancer had reached the middle and late stages of treatment, the tumors were mostly low differentiated adenocarcinoma, so this study did not carry out a detailed analysis.

### 3.3. Intraoperative Outcomes Compared between the Two Groups

The VFA-H subgroup tended to have greater intraoperative blood loss, although the relationship was not statistically significant. The BMI-H subgroup presented the same phenomenon. There was no statistical significance between the two groups regarding the number of harvested lymph nodes (*p* > 0.050). However, in the comparison of patients who underwent laparoscopic resection only, it was found that the operation time was more than that of the VFA-L group (*p* < 0.050). No similar results were found in the BMI group (*p* = 0.299), as shown in Tables 3 and 4.

### 3.4. Postoperative Outcomes Compared between the Two Groups

The incidences of postoperative complications of the VFA-H group were significantly higher than those of the VFA-L group (31.03% and 5.17%, respectively; *p* < 0.001). The incidence of diarrhea obviously increased (*p* = 0.027). In our ward, there was a low correlation between prophylactic antibiotics and bowel movement, and there was no statistical significance (|*r*| < 0.4, *p* > 0.050), as shown in Table 5. Compared to the VFA-L group, the VFA-H group had longer postoperative hospital stay (12.78 ± 11.18 and 9.28 ± 4.19, respectively; *p* = 0.027), and later first exhaust time (88.13 ± 14.04 and 81.82 ± 16.25, respectively; *p* = 0.018), but these differences were not seen in BMI (*p* > 0.050), as shown in Tables 6 and 7.

## 4. Discussion

BMI and VFA are indicators of obesity. However, BMI may not accurately reflect the intra-abdominal fat area because it is calculated by the weight divided by the square of the height of the body, but VFA may reflect the fat volume in the upper abdomen as well as in the lower abdomen. Elevated VFA can increase the difficulty of laparoscopic-assisted gastrectomy, increasing the risks of postoperative complications and prolonging postoperative recovery. A large accumulation of visceral adipose tissue is very easy to destroy and leads to a poor visual operative field. Adipose tissue easily bleeds when it is stripped and thus makes surgery more difficult [17]. When the sixth, seventh, eighth, ninth, and eleventh groups of lymph nodes were dissected from the upper and lower margins of the pancreas by stripping its adipose tissue during surgery particularly, it increased the chances of bleeding and pancreatic trauma [8]. In 17 cases of conversion to open surgery, the number of cases of the VFA-H group was significantly higher than that of the VFA-L group, but no similar conclusion was obtained in the BMI group, suggesting that VFA may increase the difficulty of laparoscopic surgery [18]. The literature has reported that the accumulation of visceral adipose tissue easily causes an increase of blood loss during surgery, which affects the safety of the operation. Although the VFA-H and BMI-H subgroups tended to have greater intraoperative blood loss, this study did not find a significant increase in intraoperative bleeding in patients in the VFA-H and BMI-H subgroups. However, through statistical analysis of patients undergoing only laparoscopic surgery, we found that the VFA-H group required longer operation time than the VFA-L group. Visceral fat area increases the difficulty of laparoscopic surgery. The results indicated that there was no difference between the two groups in the number of removed lymphatic nodes, but the average number of lymph node dissections in the two groups exceeded 25. This may be due to the amplification effect of the laparoscopic procedure and surgery by the same group of experienced surgeons, thus avoiding the effects of obesity on the operation.

These results suggest that the evaluation of the difficulty of laparoscopic-assisted gastrectomy and the risk of postoperative complications with VFA was more accurate than that of BMI. The VFA-H group compared to the VFA-L group had a greater proportion of overweight, higher postoperative complication rates, and more incidences of diarrhea. However, no similar results were found in the BMI group. Postoperative diarrhea may be related to postoperative fasting and a liquid diet, leading to the slow recovery of gastrointestinal function and antibiotic application caused by intestinal bacterial colony disorder. However, we found no correlation between prophylactic antibiotics and bowel movement by statistical analysis of data from our wards. There are few reports on the relationship between visceral fat accumulation and postoperative diarrhea. In addition, patients in the VFA-H group had longer hospital stays and later first exhaust time, which indicated that the increased VFA would delay the postoperative recovery of patients. Although postoperative complications were not significantly different in the BMI subgroup, the rate of incision infection in the BMI-H group was significantly higher than that in the BMI-L group. This may be due to the fact that subcutaneous adipose tissue is prone to liquefaction leading to wound infection.

In summary, the evaluation of the difficulty of laparoscopic-assisted gastrectomy and the risk of postoperative complications with VFA was more accurate than that of BMI. Increased VFA is a negative factor for laparoscopic-assisted gastrectomy. It increases the difficulty of laparoscopic surgery and the risk of postoperative complications and slows the postoperative recovery of patients.

## Figures and Tables

**Table 1:** Comparison of features between the low-VFA group and high-VFA group (cases (%)).

Clinicopathological features	VFA-L group (*n* = 58)	VFA-H group (*n* = 58)	*p* value
Male	45 (77.59)	44 (75.86)	0.826
BMI > 25 kg/m^2^	7 (12.07)	39 (67.24)	<0.001
Age^∗^	54.43 ± 12.47	59.50 ± 9.32	0.015
Location of tumor			0.739
Sinuses ventriculi	33 (56.90)	31 (53.45)	
Gastric body	10 (17.24)	8 (13.79)	
Gastric fundus	2 (3.45)	4 (6.90)	
Gastric antrum and body^∗∗^	10 (17.24)	13 (22.41)	
Gastric body and fundus^∗∗∗^	2 (3.45)	2 (3.45)	
Total gastric lesion	1 (1.72)	0 (0.00)	
Size of tumor (cm)^∗^	3.63 ± 2.54	3.03 ± 1.96	0.158
Operation mode			0.182
Distal gastrectomy	50 (86.21)	51 (87.93)	
Proximal gastric resection	1 (1.72)	4 (6.90)	
Total gastrectomy	7 (12.07)	3 (5.17)	
Pathological stage^a^			0.257
I	24 (41.38)	34 (58.62)	
II	16 (27.59)	11 (18.97)	
III	15 (25.86)	12 (20.69)	
IV	3 (5.17)	1 (1.72)	

^∗^Data are mean ± SD. ^∗∗^Junction of gastric antrum and body. ^∗∗∗^Junction of gastric body and fundus. ^a^Pathological stage of the tumor. VFA visceral fat area, BMI body mass index.

**Table 2:** Comparison of features between the low-BMI group and high-BMI group (cases (%)).

Clinicopathological features	BMI-L group (*n* = 70)	BMI-H group (*n* = 46)	*p* value
Male	55 (78.57)	34 (73.91)	0.561
VFA > 100 cm^2^	19 (27.14)	39 (84.78)	<0.001
Age^∗^	54.83 ± 11.87	60.22 ± 9.45	0.011
Location of tumor			0.456
Sinuses ventriculi	41 (58.57)	32 (69.57)	
Gastric body	13 (18.57)	7 (15.21)	
Gastric fundus	1 (1.43)	2 (4.35)	
Gastric antrum and body^∗∗^	13 (18.57)	4 (8.70)	
Gastric body and fundus^∗∗∗^	1 (1.43)	1 (2.17)	
Total gastric lesion	1 (1.43)	0 (0.00)	
Size of tumor (cm)^∗^	3.38 ± 2.42	3.24 ± 2.08	0.735
Operation mode			0.243
Distal gastrectomy	63 (90.00)	41 (89.13)	
Proximal gastric resection	1 (1.43)	3 (6.52)	
Total gastrectomy	6 (8.57)	2 (4.35)	
Pathological stage^a^			0.097
I	31 (44.29)	27 (58.70)	
II	17 (24.29)	7 (15.21)	
III	18 (25.71)	12 (26.09)	
IV	4 (5.71)	0 (0.00)	

^∗^Data are mean ± SD. ^∗∗^Junction of gastric antrum and body. ^∗∗∗^Junction of gastric body and fundus. ^a^Pathological stage of the tumor.

**Table 3:** Comparison of the intraoperative outcomes between the low-VFA group and high-VFA group carrying out without conversion to laparotomy.

	VFA-L group (*n* = 58)	VFA-H group (*n* = 58)	*p* value
Operation time (min)^∗^	214.53 ± 47.61	245.66 ± 98.72	0.033
Blood loss^b^ (ml)^∗^	101.29 ± 68.80	113.62 ± 70.68	0.343
Number of lymph nodes^c^^∗^	29.10 ± 10.46	28.64 ± 12.08	0.825

^∗^Data are mean ± SD. ^b^Intraoperative blood loss. ^c^Number of dissected lymph nodes.

**Table 4:** Comparison of the intraoperative outcomes between the low-BMI group and high-BMI group carrying out without conversion to laparotomy.

	BMI-L group (*n* = 70)	BMI-H group (*n* = 46)	*p* value
Operation time (min)^∗^	225.81 ± 64.22	236.61 ± 97.18	0.472
Blood loss^b^ (ml)^∗^	99.07 ± 66.54	120.22 ± 73.20	0.110
Number of lymph nodes^c^^∗^	30.36 ± 11.21	26.61 ± 11.04	0.079

^∗^Data are mean ± SD. ^b^Intraoperative blood loss. ^c^Number of dissected lymph nodes.

**Table 5:** Comparison of the postoperative outcomes between the low-VFA group and high-VFA group (cases (%)).

	VFA-L group (*n* = 58)	VFA-H group (*n* = 58)
First defecation time	*r* ^#^ = 0.109	*r* ^#^ = −0.063
*p* value	0.415	0.641

^#^Correlation coefficient.

**Table 6:** Comparison of the postoperative outcomes between the low-VFA group and high-VFA group (cases (%)).

	VFA-L group (*n* = 58)	VFA-H group (*n* = 58)	*p* value
Complication	3 (5.17)	18 (31.03)	<0.001
Pancreatic leakage	0 (0.00)	2 (3.45)	0.496
Diarrhea	0 (0.00)	6 (10.34)	0.027
Intra-abdominal hemorrhage	1 (1.72)	1 (1.72)	>0.999
Gastroplegia	1 (1.72)	1 (1.72)	>0.999
Infection of incisional wound	0 (0.00)	3 (5.17)	0.243
Pulmonary infection	1 (1.72)	3 (5.17)	0.618
Abdominal infection	0 (0.00)	2 (3.45)	0.496
First exhaust time (h)^∗^	81.82 ± 16.25	88.13 ± 14.04	0.018
Length of hospital stay^e^ (d)^∗^	9.28 ± 4.19	12.78 ± 11.18	0.027

^∗^Data are mean ± SD. ^e^Postoperative length of hospital stay.

**Table 7:** Comparison of the postoperative outcomes between the low-BMI group and high-BMI group (cases (%)).

	BMI-L group (*n* = 70)	BMI-H group (*n* = 46)	*p* value
Complication	13 (16.67)	17 (30.91)	0.053
Pancreatic leakage	2 (2.56)	0 (0.00)	0.511
Diarrhea	2 (2.56)	5 (9.09)	0.125
Intra-abdominal hemorrhage	2 (2.56)	0 (0.00)	0.511
Gastroplegia	0 (0.00)	2 (3.64)	0.169
Infection of incisional wound	1 (1.28)	3 (5.45)	0.306
Pulmonary infection	4 (5.13)	6 (10.91)	0.317
Abdominal infection	2 (2.56)	1 (1.82)	>0.999
First exhaust time (h)^∗^	83.59 ± 15.55	87.45 ± 14.98	0.154
Length of hospital stay^e^ (d)^∗^	10.09 ± 7.70	12.46 ± 9.69	0.146

^∗^Data are mean ± SD. ^e^Postoperative length of hospital stay.
